# Layering‐Triggered Delayering with Exfoliated High‐Aspect Ratio Layered Silicate for Enhanced Gas Barrier, Mechanical Properties, and Degradability of Biodegradable Polymers

**DOI:** 10.1002/gch2.202000030

**Published:** 2020-05-27

**Authors:** Jian Zhu, Anil Kumar, Pin Hu, Christoph Habel, Josef Breu, Seema Agarwal

**Affiliations:** ^1^ Macromolecular Chemistry II Bavarian Polymer Institute University of Bayreuth Universitätsstraße 30 Bayreuth 95440 Germany; ^2^ Bavarian Polymer Institute and Inorganic Chemistry University of Bayreuth Universitätsstraße 30 Bayreuth 95440 Germany

**Keywords:** biodegradable polymers, gas barrier, nanocomposites, polylactide plastic waste management

## Abstract

Research on biodegradable polymers with the intention of fast, complete degradation in industrial compost (i‐compost) for organic recyclability is paramount to identifying solutions to the problem of excessive plastic waste originating specifically from packaging. Conventional biodegradable polymers, such as polylactide (PLA), are far from optimum for this application due to the poor gas barrier properties and slow degradation. In the paper, a new concept (triggered degradation by delayering) is shown in which exfoliated, self‐assembled sodium‐hectorite (Hec) arranged in a layer‐by‐layer manner alternating with electrospun hot‐pressed PLA provides strong gas barrier properties at high humidity and simultaneously accelerates the degradation of PLA, as tested in an enzymatic solution and i‐compost. A thin composite film (thickness 56 µm) shows a tensile strength and modulus 58 and 2000 MPa, respectively, whereas oxygen permeability is as low as 0.0064 cm^3^ cm m^−2^ day^−1^ bar^−1^. Furthermore, the delayering of the composite film by swelling of Hec layer led to accelerated degradation of PLA, as shown in detail by enzymatic and compost degradation. Since such concepts for enhanced degradability are urgently needed for sustainable utilization of biodegradable polymers in plastic waste management, the present work is an important step ahead.

## Graphical abstract

Enhanced degradability, modulus, and decreased oxygen permeability required for packaging is achieved in polylactide (PLA) by making its composites with exfoliated sodium hectorite (Hec) in a layer‐by‐layer structure. The layered structure enables improved mechanical and gas permeation properties, while the delayering of composite films due to the swelling of Hec provide accelerates degradation.

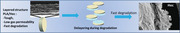

## 1 Introduction

Significant efforts have been required to solve environmental problems created by plastic pollution by expanding the application of biodegradable polymers in recent years, especially in the field of food packaging. Most of the plastic waste in landfills originates from plastic packaging. Polylactide (PLA) is often considered as a promising commercial biodegradable polymer alternative to replace petro‐based polyethylene terephthalate (PET) or polyolefins for packaging applications because of its acceptable mechanical properties, good processability and renewable agricultural source.^[^ ^1^, ^2^, ^3^, ^4^ ^]^ However, some important issues, such as toughness, heat distortion temperature and gas barrier properties, are still needed to be enhanced before it can compete with conventional plastics.^[^ ^5^, ^6^ ^]^ From a biodegradability point‐of‐view, PLA is not suitable for sustainable organic recyclability (biodegradation to carbon dioxide, water, and biomass) via industrial composting due to its slow degradation. Therefore, property modifications are required to make PLA suitable for sustainable packaging applications.

Compared with copolymerization and blending, the use of nanofillers is more effective to improve the mechanical and gas barrier properties of the PLA matrix.^[^ ^6^, ^7^, ^8^, ^9^ ^]^ Nanosized layered silicates are the most popular fillers for gas barrier enhancement for its large aspect ratio and gas impermeability which have been investigated in a wide range of research works.^[^ ^8^, ^9^ ^]^ A variety of processing strategies, including melt extrusion, in situ polymerization and solution casting (doctor blading and spin coating), have been explored for the purpose of the inclusion of impermeable layered silicate clays into polymers.^[^ ^6^, ^9^, ^10^, ^11^, ^12^, ^13^, ^14^ ^]^ However, it is always a challenge to get completely exfoliated hydrophilic‐layered silicate nanofillers into the hydrophobic polymer matrix using state‐of‐the‐art processing methods and the amount of filler is also limited due to its high viscosity.^[^ ^8^, ^15^ ^]^ Moreover, the achievement of strong interaction between the layered silicates nanoplatelets with the polymer matrix is another crucial issue.^[^ ^9^, ^16^ ^]^ Modification of the layered silicates surface with surfactant decreases the incompatibility and facilitates the delamination of layered silicates in the matrix in some cases.^[^ ^16^, ^17^ ^]^ However, the achievement of high filler content (>50 wt%) and oriented composite materials is still unworkable with the traditional techniques. According to Cussler's suggestion, the barrier improvement is nonlinearly dependent upon both the aspect ratio (α) of the filler platelets and filler content (ϕ) once the nanoplatelets are well‐dispersed and exfoliated in the polymer matrix and the orientation of each platelet's largest dimension perpendicular to the direction of the diffusion path.^[^ ^18^, ^19^ ^]^

Synthetic layered silicate (such as sodium hectorite: Hec), with a high aspect ratio, large cation‐exchange capacity and swelling properties, has been proven effective for gas barrier property enhancement by Breu and co‐workers and has attracted increasing attention in recent years.^[^ ^20^, ^21^, ^22^ ^]^ In order to attain a nanocomposite to conform to Cussler's theory, a new approach of coating a high volume fraction filler nanocomposite layer consisting mainly of single (exfoliated) layered silicate platelets onto the surface of the polymer substrate shows great advantages in the decreasing of the gas permeability. The typical example of coating layered silicates is layer‐by‐layer assembly of negative‐charged clay and positive‐charged polyelectrolyte onto the surface of the substrate, utilizing an electrostatic attraction effect.^[^ ^23^, ^24^, ^25^ ^]^ More than 90 % of the reduction of oxygen permeability of PLA film was observed by Svagan et al. when 40 bilayers of chitosan/montmorillonite coating at 50% relative humidity (RH) were used.^[^ ^23^ ^]^ We recently studied gas permeability of PLA by spray‐coating a glycol chitosan‐Hec layer onto the surface of the PLA films.^[^ ^26^ ^]^ The oxygen transmission rate (OTR) of the resultant film with a coating of 1.4 µm shows a reduction compared to the neat PLA substrate (874.2 cm^3^ m^−2^ day^−1^ bar^−1^, thickness 25 µm) foil by four orders of magnitude to 0.08 cm^3^ m^−2^ day^−1^ bar^−1^ (50% RH), which outperforms OTR of neat polyethylene terephthalate by a factor of ≈400.^[^ ^26^ ^]^ However, both layer‐by‐layer assembling and spray coating techniques are time‐consuming to obtain a valuable thickness of the filler by water removal and repeated cyclic coating.^[^ ^23^, ^26^, ^27^ ^]^ Consideration of the high porosity of an electrospun fiber mat,^[^ ^28^, ^29^, ^30^ ^]^ a new fast and scalable approach of coating layered silicate onto the surface of an electrospun fiber mat with a filtration through method and, subsequently, hot‐pressed into transparent film is a promising approach.^[^ ^27^ ^]^

In this work, we prepared layered composite films with alternating layers of PLA and compact Hec‐poly(vinyl pyrrolidone) (PVP) (Hec exfoliated with PVP (PVP‐Hec; Hec‐layer)) with an excellent gas‐barrier, mechanical properties, and enhanced degradability using this approach. Electrospinning of PLA followed by formation of a compact Hec thin layer on it by vacuum assisted filtration of Hec‐dispersion provided PLA‐coated Hec. Hot‐pressing of different numbers of electrospun PLA‐coated Hec provided lightweight layered composite films, thickness ranging from 25 to 56 µm. The preparation method has the advantage that the composite films can be made in a modular way by changing the number of PLA‐coated Hec layers, Hec‐ and PLA‐layer thickness. The Hec and PLA‐layer thickness can be changed by changing the Hec concentration in dispersion or by filtering different volumes of the Hec‐dispersions and changing the time of electrospinning of PLA, respectively in a simple way. In this method, highly porous electrospun PLA acted both as matrix polymer and the filter for coating of Hec by filtration‐through method. The method is not applicable to nonporous melt‐blown or solvent casted PLA films as matrix material. In such a case, the exfoliated Hec should be coated by spray coating. Interestingly, the layered structure not only provided an enhanced gas‐barrier and mechanical properties but also a new and desired concept of accelerating the degradation of PLA, as tested in enzymatic solution and i‐compost. The enhanced degradation is due to water‐triggered delayering of the composite films most probably by swelling of the intermediate Hec in the composite films, exposing thinner PLA layers with a high surface area for degradation. Detailed studies regarding the preparation of composite films, their structural, mechanical characterization and degradation behavior is reported.

## 2 Results and Discussions

**Scheme 1:**
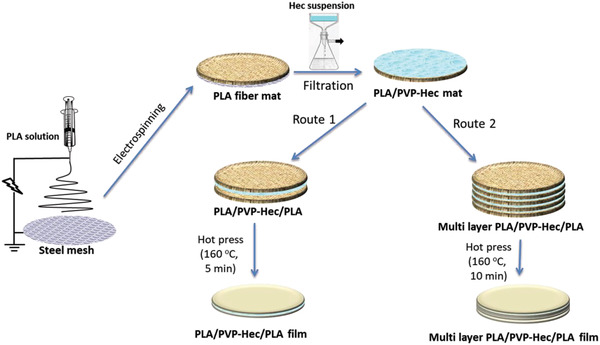
Fabrication process of PLA/Hec composite films.

**Figure 1:**
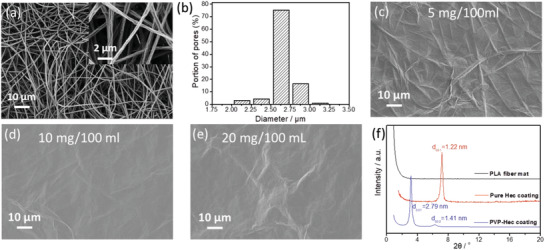
a) Scanning electron microscope (SEM) images of electrospun PLA fibers and b) pore size distribution of PLA fiber mat. c–e) SEM images of the PVP‐Hec coated onto the surface of PLA mat when the amount of Hec in the suspension is c) 5 mg, d) 10 mg, and e) 20 mg. f) XRD patterns of PLA fiber mat, pure Hec coating and the PVP‐Hec layered PLA fiber mat (prepared from 5 mg/100 mL Hec suspension).

Multilayered composite films of PLA with exfoliated Hec were prepared, as shown in **Scheme** **1**. Electrospun PLA fiber mat used for the filtration of PVP‐Hec showed a pore size distribution of 2–3.5 µm with a maximum number of pores around 2.6 µm, as shown in **Figure** **1**a,b. The fiber diameter was in the range of 400–650 nm (Figure 1a). After vacuum filtration, the PVP‐Hec platelets were homogenously deposited on the surface of the PLA fiber mat (Figure 1c–e). The smectic crystal structure of PVP‐Hec layered on the PLA mat was observed by X‐ray diffraction (XRD) (Figure 1f). After filtration of PVP‐Hec, a d‐spacing of 2.79 nm (d*_002_* at 1.41 nm) is present in the system. For comparison, also the neat Hec coating is shown in Figure 1f, showing a d‐spacing of 1.22 nm, describing mono‐hydrated Hec nanoplatelets. As this peak is absent in the PVP‐Hec coating it can be concluded that the single Hec nanoplatelets are completely separated by PVP volume, describing a mono‐hydrated Hec structure. There was no sharp interference observed from the PLA.

**Figure 2:**
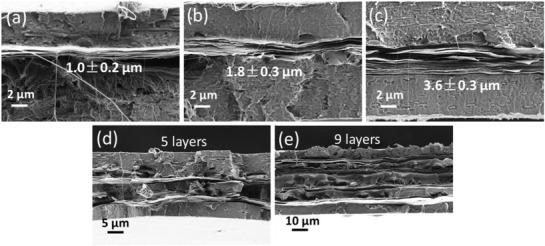
SEM images of the cross‐section of three layered composite films from different amounts of Hec in 100 mL suspension: a) 5 mg, 3L‐PLA/PVP‐Hec‐5, b) 10 mg, 3L‐PLA/PVP‐Hec‐10, and c) 20 mg, 3L‐PLA/PVP‐Hec‐20. SEM images of cross‐section of d) 5 layers (5L‐PLA/PVP‐Hec‐10) and e) 9 layers (9L‐PLA/PVP‐Hec‐10) composite films from 10 mg/100 mL Hec suspension.

The multilayered laminar composite films were made by hot‐pressing different numbers of bilayer PVP‐Hec self‐assembled on electrospun PLA mats. The laminar composite films with the PVP‐Hec layers sandwiched between PLA layers were designated as *n*L‐PLA/PVP‐Hec‐*x*, where *n* is the total number of layers and *x* is the amount of Hec in the suspension used for preparing the films. The thickness of the PVP‐Hec layer was increased from 1 to 3.6 µm as observed from the cross‐section in scanning electron microscope (SEM) (**Figure** **2**a–c) when the amount of Hec increased from 5 to 10 and 20 mg (3L‐PLA/PVP‐Hec‐5, 3L‐PLA/PVP‐Hec‐10, and 3L‐PLA/PVP‐Hec‐20, respectively) in water suspension (100 mL suspension used every time). The actual Hec content in composite films was determined by thermogravimetric analysis (TGA) (residue left at 800 °C) and was found to be ≈7%, 11%, and 15% for 3L‐PLA/PVP‐Hec‐5, 3L‐PLA/PVP‐Hec‐10, and 3L‐PLA/PVP‐Hec‐20, respectively (Figure S1 and Table S1, Supporting Information). The total thickness of the final layered composite films is in the range of 25–56 µm based on the different number of layers (Table S1, Supporting Information). Multilayered PLA/PVP‐Hec composite films morphologies were also obvious from the SEM pictures (Figure 2d–f).

**Figure 3:**
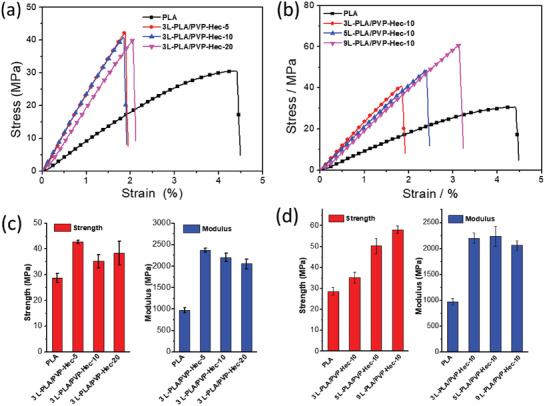
Stress‐strain curves and statistical results of mechanical testing of three‐layered and multilayered PLA/PVP‐Hec composite films.

A PVP‐Hec sandwich layer between two PLA films greatly enhanced more than twice the elastic modulus and a slight increase in the mechanical strength of the PLA, as observed from stress‐strain tests shown in **Figure** **3**a and Table S1 (Supporting Information). Almost no effect of the amount of Hec on strength and modulus of composite films was seen (Figure 3a–c). The Hec‐layer in contact with PLA acts as stress‐transfer agent and hence increased, in general the tensile strength in composite films. Further, the increased amount of Hec adds to the thickness by layer‐by‐layer assembly but do not change the contact surface with PLA matrix leading to almost similar tensile strength and modulus.

Interestingly, the tensile strength rises gradually to about 58.1 MPa for the multilayer composite film (9L‐PLA/PVP‐Hec‐10) (Hec content 16.5 wt%) in comparison to PLA (30 MPa) (Figure 3b). This value is about 65% higher than the three‐layered composite membrane (3L‐PLA/PVP‐Hec‐10) and surpasses the pure PLA film by 94% (Figure 3c,d). Furthermore, the composite films were flexible, as shown in the bending test in Figure S2 (Supporting Information); even the multilayer samples (sample 9L‐PLA/PVP‐Hec‐10 shown as representative example) could bear at least 100 times of bending and did not develop any cracks.

**Figure 4:**
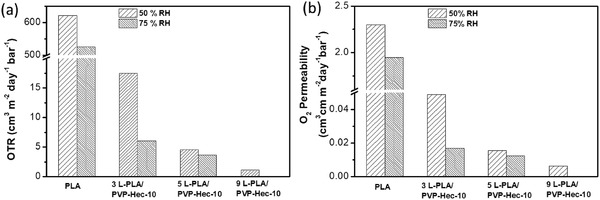
Oxygen transmission rate (OTR) a) and O_2_ permeability b) of PLA/PVP‐Hec composite films.

For the applications the neat oxygen transmission rates (OTRs) are of prime importance. PLA without PVP‐Hec layer showed OTRs of 621.2 and 525.4 cm^3^ m^−2^ day^−1^ bar^−1^ at 50% and 75% RH, respectively. As can be seen in **Figure** **4**a a 1.8 µm PVP‐Hec layer sandwiched between two PLA films (sample 3L‐PLA/PVP‐Hec‐10, thickness of 28 µm) already led to a reduction of OTR by 97.2% and 98.9% with resulting OTRs as small as 17.5 and 6.05 cm^3^ m^−2^ day^−1^ bar^−1^, respectively. For a better comparison of the barrier properties of samples with different thicknesses, the oxygen permeability (OP) has to be calculated. For this purpose, the experimentally determined OTR is multiplied with the overall thickness of the samples (Table S1, Supporting Information) and afterward normalized to a certain thickness, here cm. As Figure 4b is showing, the OPs are reduced significantly in dependency of the number of PVP‐Hec layers up to a factor of >3.5 × 10^2^ to a resulting OP of 0.0064 cm^3^ cm m^−2^ day^−1^ bar^−1^ (9L‐PLA/PVP‐Hec‐10, 50% RH). This OP value is much smaller than PLA films coated with chitosan/montmorillonite using a layer‐by‐layer technique, and outperform some of the state‐of‐the‐art packaging materials,^[^ ^31^ ^]^ like PET (>0.1 cm^3^ cm m^−2^ day^−1^ bar^−1^).^[^ ^23^ ^.^ ^32^ ^]^ The barrier properties can be affected by environmental conditions like relative humidity (RH).^[^ ^33^ ^]^ Therefore, we measured both 50%RH and 75%RH to show barrier performance at application‐oriented conditions for packaging (Figure 4b). The improvement of the O_2_ barrier property was attributed to the highly compact Hec lamellar microstructure of the PVP‐Hec layer. The large lateral dimension and high aspect ratio of the Hec nanoplatelets extended the diffusion pathway (tortuous path) of the gas molecules and thereby enhanced the gas barrier properties.

**Figure 5:**
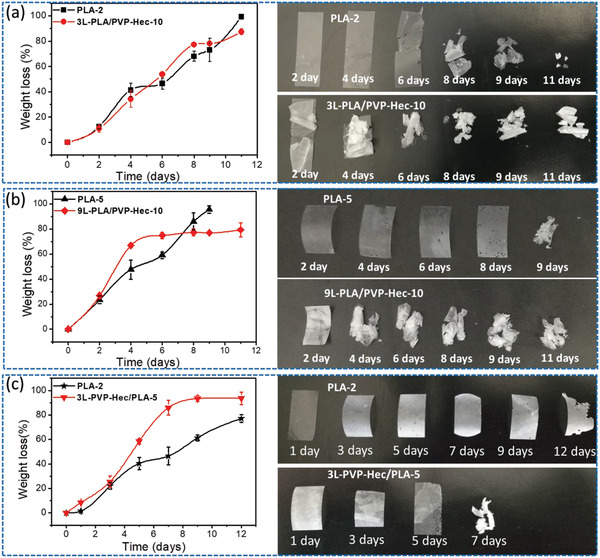
Weight loss change and the appearance change of three groups comparing PLA and PLA/PVP‐Hec composite films.

Fragmentation by hydrolysis of the ester units in the PLA backbone is the first step of biodegradation.^[^ ^34^, ^35^ ^]^ Therefore, the effect of clay and composite film morphology on PLA fragmentation was studied in controlled tests using Proteinase K enzyme and i‐compost. The PLA films were made by hot‐pressing different number of electrospun PLA mats, as used in multilayered composite films, to make the results more comparative. The nine layered sample 9L‐PLA/PVP‐Hec‐10, for example, contains five layers of PLA and four layers of PVP‐Hec, so that the corresponding PLA film used for comparison purpose was fabricated by hot‐pressing five PLA electrospun mats together and designated as PLA‐5. In the same way, PLA‐2 is prepared by hot‐pressing two electrospun PLA membranes to provide a realistic comparison to 3L‐PLA/PVP‐Hec‐10 and 3L‐PVP‐Hec/PLA‐5. Based on an enzymatic degradation test, the first important fact was that the presence of PVP‐Hec layers accelerated the enzymatic degradation of the matrix PLA, as revealed from the weight loss curves and visual observations (**Figure** **5**). This fact was very obvious from results of multilayered composite films with a total of nine layers (9L‐PLA/PVP‐Hec‐10) in which each PLA layer alternated with a PVP‐Hec layer. More than 40 wt% of pure PLA‐5 remained after 6 days of degradation. The weight loss of sample 9L‐PLA/PVP‐Hec‐10 (PLA: PVP‐Hec 76:24 weight ratio) showed almost complete fragmentation of PLA in 6 days. It showed more than 90% degradation in 4 days. The weight loss for 9L‐PLA/PVP‐Hec‐10 is around 76 wt% in six days and did not change further implying complete degradation, as seen in Figure 5b. This value corresponds very well with TGA result that shows about 24% PVP‐Hec in the sample (Figure S1, Supporting Information). The original composition of 9L‐PLA/PVP‐Hec‐10 is about PLA: PVP‐Hec 76:24 weight ratio.

**Figure 6:**
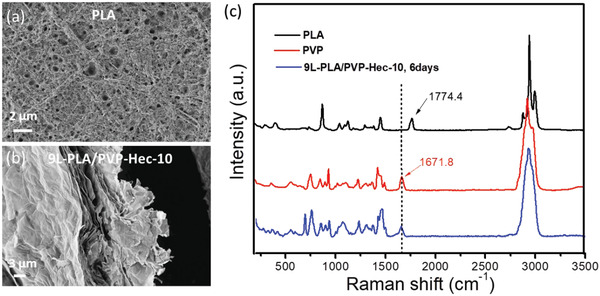
a) SEM images of PLA and b) nine‐layered PLA/PVP‐Hec composite films after 6 days of enzyme degradation. c) Raman spectrum of pure PLA, PVP and the residual of 9L‐PLA/PVP‐Hec‐10 after 6 days of enzyme degradation.

To further confirm this, morphologies of PLA residue from pure PLA film and the residue of 9L‐PLA/PVP‐Hec‐10 after 6 days were compared (**Figure** **6**a). The residue of 9L‐PLA/PVP‐Hec‐10 showed very compact sheet‐type morphology, which looks like Hec, whereas the residue of PLA showed a porous structure. Furthermore, the Raman spectra of pure PLA, PVP and the residue were measured to prove that the residue was PVP‐Hec after 6 days of enzymatic degradation and the absence of any traces of PLA in the residue (Figure 6b). The characteristic peak of the carbonyl band of PLA at 1774 cm^−1^ disappeared after 6 days in the residue, whereas the peak at 1672 cm^−1^ belonging to PVP was still existent, showing that the residue was nothing but PVP‐Hec (five points of the residue tested randomly) (Figure 6c).

**Figure 7:**
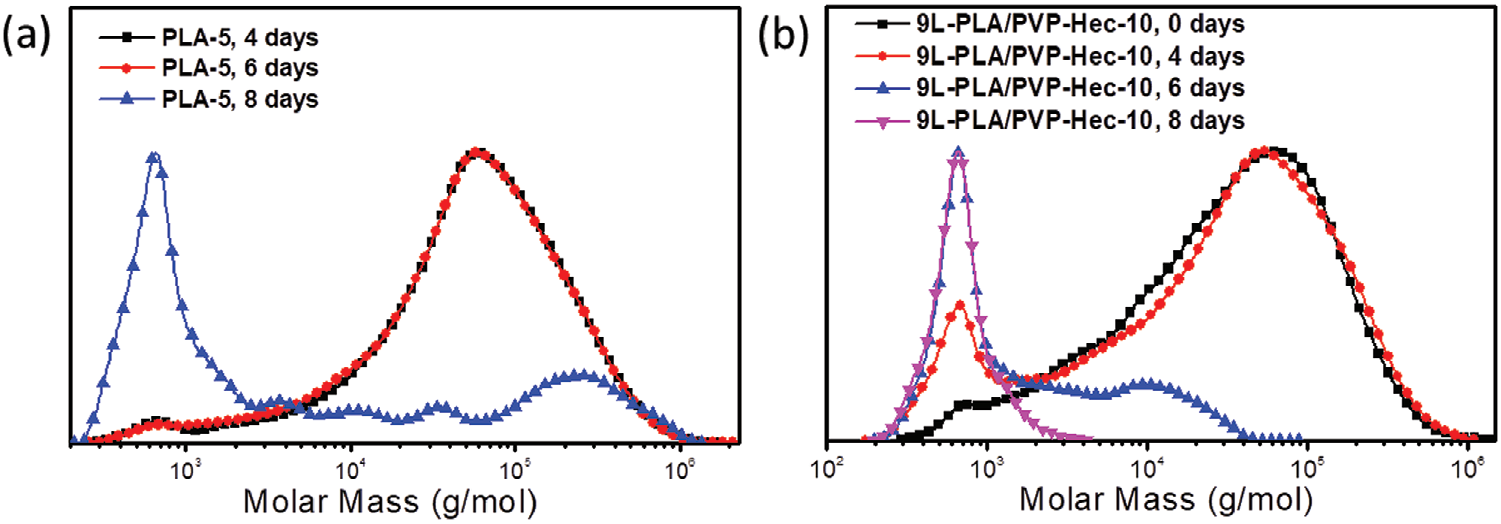
Gel‐permeation chromatography (GPC) profiles of a) PLA‐5 and b) 9L‐PLA/PVP‐Hec‐10 films after enzyme degradation for different days.

The nanocomposites prepared by melt‐extrusion of PLA and appropriate layered silicates are known to enhance the hydrolysis of PLA due to the water absorption and heterogenous catalysis of ester hydrolysis.^[^ ^36^, ^37^, ^38^ ^]^ The composite films of 9L‐PLA/PVP‐Hec‐10 have pure hydrophobic PLA as the outside (top and bottom) film surfaces. The scenario regarding the exposure of films to water should be same as that of pure PLA film. In spite of this, a clear positive effect of Hec on PLA degradation was observed. The layered morphology of the composite films provided an advantage for degradation that it delayered to individual layers in contact with enzyme/buffer solution after almost 2 days (Figure S3, Supporting Information). This led to the exposure of several PVP‐Hec and much thinner PLA surfaces to enzyme solution instead of two hydrophobic PLA surfaces of the original composite film enhancing the degradation rate. Each individual layer of PLA in 9L‐PLA/PVP‐Hec‐10 is about 9–10 µm. The residue after different intervals of time during enzymatic degradation was dissolved in chloroform and the change in the molar mass was followed by gel permeation chromatography (GPC). The PLA showed degradation by surface erosion as the molar mass of the PLA remained the same after 4 and 6 days of degradation in spite of significant changes in weight during this time (**Figure** **7**a). In the case of 9L‐PLA/PVP‐Hec‐10, the molar mass change was only obvious in the low molar mass region after 4 days of degradation (Figure 7b). The residue showed an increase in the lower mass fraction at peak molar mass (Mp) = 670 with no significant change in Mp (61 000) in the high molar mass region (we do not mention here the number of average/molecular weight averages as the peaks are multimodal) in spite of a weight loss. After 6 days, there was a significant shift in the Mp from 61 000 to 9800 in the high molar mass region, whereas the intensity at low molar mass (Mp = 660) increased significantly. After 8 days, the residue did not show any traces of high molar mass fractions, which is in line with the weight loss. The GPC results give a hint of the surface erosion mechanism similar to that of PLA.

The experiment regarding enzymatic degradation carried out with three‐layer composite films comprising PVP‐Hec sandwiched between 2 PLA surfaces (3L‐PLA/PVP‐Hec‐10; PLA: PVA‐Hec = 82: 18 weight ratio) and PLA with PVP‐Hec on both surfaces (3L‐PVP‐Hec/PLA‐5) in comparison to the corresponding PLA film supported the conclusion drawn with nine layered composite films. The 3L‐PLA/PVP‐Hec‐10 showed slightly faster degradation than PLA‐2 (Figure 5a), whereas 3L‐PVP‐Hec/PLA‐5 showed the significant effect of Hec in enhancing the degradation (Figure 5c).

**Figure 8:**
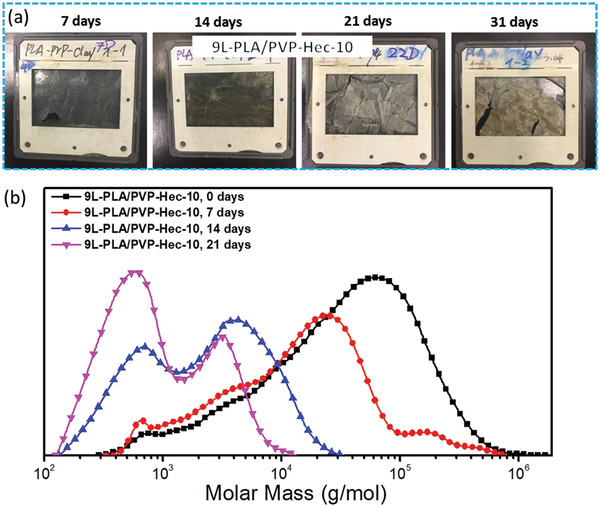
a) Photos of 9L‐PLA/PVP‐Hec‐10 films at different time intervals in compost. b) GPC profiles of 9L‐PLA/PVP‐Hec‐10 films at different time intervals

Compostability of the polymer materials is an important method to evaluate the organic recyclability which we carried out by burying composite membranes fixed onto polyethylene frames in i‐compost at 60 °C. The qualitative analysis by the observation of appearance of the buried films at different time intervals as illustrated in **Figure** **8**a where fragmented sheets are shown. The fragments were dissolved in chloroform and tested for molar mass by GPC (Figure 8b). There was already a significant change in the Mp after 7 days of burial in compost with several obvious low molar mass fragments. The Mp kept on shifting to lower molar mass with time with the appearance of new peaks at lower molar mass with the broadening of molar mass distributions (Figure 8b). In 21 days, the GPC curve showed only low molar peaks, which were very similar to the extract from blank compost (without any buried polymer), indicating almost completed degradation.

## 3 Conclusions

Mechanically strong, flexible, extremely low oxygen permeability and excellent degradation performances have been achieved by developing a strategy of making a composite film (25–56 µm) by alternately stacking a different number of very thin layers of PLA (about 10 µm) and PVP‐Hec (1–2 µm). The exfoliated Hec, assembled in the form of a thin layer on electrospun PLA, provided an enhanced modulus and increased tortuous path for low gas permeation even at high humidity. PVP‐Hec swells in water, therefore, the complete layered composite film structure delayered in about 48 h in contact with enzyme solution, enhancing the enzymatic degradability of the PLA. The delayering exposed many thin PLA layers and PVP‐Hec surfaces for degradation in comparison to only two hydrophobic PLA surfaces in pure PLA. The numbers of PLA surfaces available were enhanced in addition to improved hydrophilicity due to PVP‐Hec leading to fast degradability. The composite films showed promise as fast degrading material in i‐compost, in addition to also having suitable mechanical and gas barrier properties required for packaging materials.

More studies are planned for the proof of complete assimilation to carbon dioxide, water and biomass during degradation by respirometric methods and by the use of 13C‐labelled polymer. Biodegradable alternatives for PVP will also be utilized in the future. The method opens up a broad application potential of our layer‐delayer concept of biodegradable composites by enhancing the degradation with a balance of good mechanical and gas barrier properties required for packaging.

## 4 Experimental Section

##### Materials

Poly(lactic acid) (Ingeo Biopolymer PLA 4043D, Nature‐Works LLC) and poly(vinyl pyrrolidone) (PVP, MW: 360 000, Sigma Aldrich) were used as received. The Hectorite ([Na_0.5_]^inter^[Mg_2.5_Li_0.5_]^oct^[Si_4_]^tet^O_10_F_2_) (Hec) was synthesized via melt synthesis according to an established procedure from literature.^[^ ^39^ ^]^ The Hec platelets show an aspect ratio of ≈20 000. Chloroform (stabilize with 0.6% ethanol, VWR International), *N*,*N*‐dimethylformamide (DMF, 99.9%, Fisher Chemical) and acetic acid (>99.8%, Sigma Aldrich) were used without further purification.

### Electrospinning of PLA

The 7 wt% PLA solution for electrospinning was prepared by dissolving PLA in a mixture of chloroform, DMF and acetic acid (weight ratio 70:15:15). First, PLA was dissolved completely in chloroform. After this, definite amounts of DMF and acetic acid were added to the PLA solution in chloroform and stirred for 10 min. The solution was used immediately for electrospinning. A rotary circular 325‐mesh stainless steel wire mesh (diameter 6.4 cm, area 43 cm^2^) was used as the collector with the distance of 20 cm to the tip of nozzle (diameter of the nozzle is 0.4 mm). An amount of 0.5 mL of PLA solution was used each time with a voltage of 14 kV.

#### Preparation of PLA‐Hec Layer‐By‐Layer Films—PLA‐Hec Layer‐By‐Layer Mats

PLA‐Hec layer‐by‐layer mats were prepared by vacuum‐assisted filtration of PVP‐Hec suspension through PLA porous membrane made in previous section. The Hec suspension was prepared by dispersing 1 g Hec in 199 mL deionized water and stirring vigorously for 3 days to form a 0.5 wt% suspension. The PVP‐Hec suspensions with different Hec concentrations (5, 10, or 20 mg /100 mL^−1^, pure Hec excluding PVP) were obtained by adding a definite amount of PVP solution (1 wt%) and stirring for another 10 min. The mass ratios of Hec and PVP were always controlled to 6:4 (Hec: PVP)^[^ ^40^ ^]^ and 100 mL suspension was filtrated each time. Before the filtration of the PVP‐Hec dispersion, 100 mL pure deionized water was filtered to wet the PLA mat.

#### Preparation of PLA‐Hec Layer‐By‐Layer Films—Preparation of Laminar PLA/Hec Composite Films

The dried PVP‐Hec‐coated PLA mats were peeled from the steel mesh and covered with another PLA electrospun mat on the PVP‐Hec side. The tri‐layered mats (PLA/PVP‐Hec/PLA) were pressed with a load of 15 000 pounds at 160 °C for 5 min to obtain a transparent PLA/PVP‐Hec/PLA laminar film (route 1 in Scheme 1). The multilayer PLA/Hec films were obtained by stacking many PVP‐Hec‐coated bilayer PLA mats together (10 mg/100 mL Hec suspension was always used for this) and then covering with a neat PLA mat on the topside and, subsequently, hot‐pressing with 15 000 pounds at 160 °C for 10 min (route 2 in Scheme 1). The final film produced is called *n*L‐PLA/PVP‐Hec‐*x*, where *n* is the number of total layers (including PLA and PVP‐Hec layers) and *x* is the amount of Hec on the suspension each time.

Two PVP‐Hec‐coated PLA bilayer mats were stacked together from the PLA side and hot‐pressed; a film with PVP‐Hec coating on two outer sides was formed and classified as *n*L‐PVP‐Hec/PLA‐*x*.

### Characterizations

The pore size distribution of the electrospun PLA fibrous mat was tested with pore size meter (PSM 165, Topas GmbH). The morphology of the PLA mat, the PVP‐Hec and GK‐Hec deposited on the surface of the PLA mat, and the cross‐section of the pressed PLA/Hec composite films were observed by SEM (Zeiss Leo 1530). The macrostructure of the PVP‐Hec‐coated PLA mats was investigated by X‐ray diffraction measurements (XRD). XRD patterns were obtained using nickel‐filtered Cu Kα radiation (λ = 1.54187 Å) on a Bragg‐Brentano‐type diffractometer (EMPYREAN) equipped with a pixcel detector. All patterns were analyzed using Panalytical's Highscore Plus software. Mechanical performances were determined by stress–strain tests on a tensile testing machine (Zwick/Roell, BT1‐FR0.5TN.D14). The samples for the tensile measurement were cut to a size of 3 × 40 mm. The tensile speed applied to the samples was 5 mm min^−1^ and the pristine effective tensile length was 10 mm. The slope of the linear region of the stress–strain curves was used to determine the elastic modulus. All the tests for each sample were measured at least five times and statistics the average date as the final results. The contents of Hec in the final composite films were tested with TGA under air atmosphere at the temperature rising rate of 10 °C min^−1^. Raman spectrum (WITec alpha 300 RA^+^, Germany) was adopted to determine the stability of the coating layer adhering onto the PLA substrate. The wavenumber of the laser was 532 nm and the testing distance was controlled at 9.1 mm.

The OTR of the PLA/Hec composite were determined on a Mocon Ox‐Tran 2/21 (Mocon Inc. Minneapolis USA) at room temperature and 50% and 75% RH. A mixture of 95% N_2_ and 5% H_2_ (Linde Formiergas 95/5) was used as the carrier gas and 100% O_2_ (Linde Sauerstoff 3.5) as the permeant. The effective sample area was controlled to 5 cm^2^ by covering with aluminum masks (type MO025‐493, Mocon Inc.).

Enzymatic degradation phenomena were based on a previously published system^[^ ^35^ ^]^ by using proteinase K with the concentration of 0.005 mg mL^−1^ in Tris buffer/hydrochloric acid solution (pH = 8.5). The specimen with a size of 20 × 10 mm was placed in a vial filled with 3 mL proteinase K/buffer solution containing 0.2 mg mL^−1^ sodium azide (Figure S4a, Supporting Information). The enzyme/buffer solution system was changed every 24 h to retain the enzymatic activity. Three replicate specimens for each given experiment were taken out from enzyme/buffer solution and washed with distilled water three times. Finally, the specimens were vacuum dried at room temperature for 48 h and subsequently weighed to calculate the weight loss and GPC, and observed through SEM.

The specimens for compost degradation were fixed onto a plastic frame, as shown in Figure S4b,c (Supporting Information).^[^ ^41^ ^]^ The specimens were buried in i‐compost and incubated in a 60 °C oven. A mixture of mature compost was derived from an industrial composting plant in Buchstein, Germany. Each sample was repeated in parallel three times. Each specimen was taken out of the compost after 7, 14, and 22 days. The molecular weight was measured with GPC and morphology was observed with SEM.

## Conflict of Interest

The authors declare no conflict of interest.

## Supporting information

Supporting Information

## References

[1] A. Dorigato , M. Sebastiani , A. Pegoretti , L. Fambri , J. Polym. Environ. 2012, 20, 713.

[2] S. Inkinen , M. Hakkarainen , A.‐C. Albertsson , A. Södergård , Biomacromolecules 2011, 12, 523. 21332178 10.1021/bm101302t

[3] R. E. Drumright , P. R. Gruber , D. E. Henton , Adv. Mater. 2000, 12, 1841.

[4] M. D. Sanchez‐Garcia , J. M. Lagaron , Cellulose 2010, 17, 987.

[5] R. A. Auras , B. Harte , S. Selke , R. Hernandez , J. Plast. Film Sheeting 2003, 19, 123.

[6] H. Ebadi‐Dehaghani , M. Barikani , H. A. Khonakdar , S. H. Jafari , U. Wagenknecht , G. Heinrich , Polym. Test. 2015, 45, 139.

[7] H. Fukuzumi , T. Saito , T. Iwata , Y. Kumamoto , A. Isogai , Biomacromolecules 2009, 10, 162. 19055320 10.1021/bm801065u

[8] H. M. De Azeredo , Food Res. Int. 2009, 42, 1240.

[9] N. Najafi , M. Heuzey , P. Carreau , Compos. Sci. Technol. 2012, 72, 608.

[10] M. A. Paul , M. Alexandre , P. Degée , C. Calberg , R. Jérôme , P. Dubois , Macromol. Rapid Commun. 2003, 24, 561.

[11] D. A. Kunz , J. Schmid , P. Feicht , J. Erath , A. Fery , J. Breu , ACS Nano 2013, 7, 4275. 23544864 10.1021/nn400713e

[12] E. S. Tsurko , P. Feicht , F. Nehm , K. Ament , S. Rosenfeldt , I. Pietsch , K. Roschmann , H. Kalo , J. Breu , Macromolecules 2017, 50, 4344.

[13] J. Yao , S. Chen , C. Ma , G. Zhang , J. Mater. Chem. B 2014, 2, 5100. 32261844 10.1039/c4tb00545g

[14] H. Tetsuka , T. Ebina , H. Nanjo , F. Mizukami , J. Mater. Chem. 2007, 17, 3545.

[15] G. X. Chen , J. S. Yoon , Macromol. Rapid Commun. 2005, 26, 899.

[16] S. L. Phua , L. Yang , C. L. Toh , S. Huang , Z. Tsakadze , S. K. Lau , Y.‐W. Mai , X. Lu , ACS Appl. Mater. Interfaces 2012, 4, 4571. 22931194 10.1021/am300947b

[17] S. Maisanaba , N. Ortuño , M. Jordá‐Beneyto , S. Aucejo , Á. Jos , Appl. Clay Sci. 2017, 138, 40.

[18] E. Cussler , S. E. Hughes , W. J. Ward III , R. Aris , J. Membr. Sci. 1988, 38, 161.

[19] G. Choudalakis , A. Gotsis , Eur. Polym. J. 2009, 45, 967.

[20] M. W. Möller , D. A. Kunz , T. Lunkenbein , S. Sommer , A. Nennemann , J. Breu , Adv. Mater. 2012, 24, 2142. 22431395 10.1002/adma.201104781

[21] M. W. Möller , T. Lunkenbein , H. Kalo , M. Schieder , D. A. Kunz , J. Breu , Adv. Mater. 2010, 22, 5245. 20839253 10.1002/adma.201002559

[22] E. Doblhofer , J. Schmid , M. Rieß , M. Daab , M. Suntinger , C. Habel , H. Bargel , C. Hugenschmidt , S. Rosenfeldt , J. Breu , ACS Appl. Mater. Interfaces 2016, 8, 25535. 27603150 10.1021/acsami.6b08287

[23] A. J. Svagan , A. Åkesson , M. Cárdenas , S. Bulut , J. C. Knudsen , J. Risbo , D. Plackett , Biomacromolecules 2012, 13, 397. 22229499 10.1021/bm201438m

[24] M. A. Priolo , D. Gamboa , K. M. Holder , J. C. Grunlan , Nano Lett. 2010, 10, 4970. 21047123 10.1021/nl103047k

[25] M. A. Priolo , D. Gamboa , J. C. Grunlan , ACS Appl. Mater. Interfaces 2010, 2, 312.

[26] C. Habel , M. Schöttle , M. Daab , N. J. Eichstaedt , D. Wagner , H. Bakhshi , S. Agarwal , M. A. Horn , J. Breu , Macromol. Mater. Eng. 2018, 303, 1800333.

[27] J. Zhu , C. Habel , T. Schilling , A. Greiner , J. Breu , S. Agarwal , Macromol. Mater. Eng. 2019, 304, 1800779.

[28] S. Agarwal , A. Greiner , J. H. Wendorff , Prog. Polym. Sci. 2013, 38, 963.

[29] Y. Ding , H. Hou , Y. Zhao , Z. Zhu , H. Fong , Prog. Polym. Sci. 2016, 61, 67.

[30] S. Jiang , Y. Chen , G. Duan , C. Mei , A. Greiner , S. Agarwal , Polym. Chem. 2018, 9, 2685.

[31] A. Detzel , F. Bodrogi , B. Kauertz , C. Bick , F. Welle , M. Schmid , K. Schmitz , K. Müller , H. Käb , Biobasierte Kunststoffe als Verpackung von Lebensmitteln, Bundesministerium für Ernährung und Landwirtschaft (Auftraggeber), Fachagentur für Nachwachsende Rohstoffe (Projektträger), 2018, https://www.ifeu.de/wp‐content/uploads/Endbericht‐Bio‐LVp_20180612.pdf.

[32] E. S. Tsurko , P. Feicht , C. Habel , T. Schilling , M. Daab , S. Rosenfeldt , J. Breu , J. Membr. Sci. 2017, 540, 212.

[33] V. Siracusa , Int. J. Polym. Sci. 2012, 2012, 1.

[34] P. Stloukal , S. Pekařová , A. Kalendova , H. Mattausch , S. Laske , C. Holzer , L. Chitu , S. Bodner , G. Maier , M. Slouf , Waste Manage. 2015, 42, 31. 10.1016/j.wasman.2015.04.006 25981155

[35] Y. Tokiwa , B. P. Calabia , Appl. Microbiol. Biotechnol. 2006, 72, 244. 16823551 10.1007/s00253-006-0488-1

[36] M.‐A. Paul , C. Delcourt , M. Alexandre , Ph. Degée , F. Monteverde , Ph. Dubois , Polym. Degrad. Stab. 2005, 87, 535.

[37] S. S. Ray , K. Yamada , M. Okamoto , K. Ueda , Macromol. Mater. Eng. 2003, 288, 203.

[38] P. M. S. Souza , A. R. Morales , M. A. Marin‐Morales , L. H. I. Mei , J. Polym. Environ. 2013, 21, 738.

[39] M. Stöter , D. A. Kunz , M. Schmidt , D. Hirsemann , H. Kalo , B. Putz , J. Senker , J. Breu , Langmuir 2013, 29, 1280. 23286394 10.1021/la304453h

[40] Z. Wang , K. Rolle , T. Schilling , P. Hummel , A. Philipp , B. A. F. Kopera , A. M. Lechner , M. Retsch , J. Breu , G. Fytas , Angew. Chem., Int. Ed. 2020, 59, 1286. 10.1002/anie.201911546 PMC6972559 31714661

[41] H. Wang , M. Langner , S. Agarwal , Polym. Eng. Sci. 2016, 56, 1146.

