# Supplementary material for: Layering‐Triggered Delayering with Exfoliated High‐Aspect Ratio Layered Silicate for Enhanced Gas Barrier, Mechanical Properties, and Degradability of Biodegradable Polymers
Source: Glob Chall. 2020 May 27;4(9):2000030. doi: 10.1002/gch2.202000030 (PMC7507042; doi:10.1002/gch2.202000030)
Supplement: Supplementary file 1 — Supporting Information [file GCH2-4-2000030-s001.pdf]

Copyright WILEY-VCH Verlag GmbH & Co. KGaA, 69469 Weinheim, Germany, 2020.

Supporting Information

for *Global Challenges,* DOI: 10.1002/gch2.202000030

Layering-Triggered Delayering with Exfoliated High-Aspect Ratio Layered Silicate for Enhanced Gas Barrier, Mechanical Properties, and Degradability of Biodegradable Polymers

*Jian Zhu, Anil Kumar, Pin Hu, Christoph Habel, Josef Breu, and Seema Agarwal**

Copyright WILEY-VCH Verlag GmbH & Co. KGaA, 69469 Weinheim, Germany, 2020.

Supporting Information

Layering-Triggered Delayering with Exfoliated High-Aspect Ratio Layered Silicate for Enhanced Gas Barrier, Mechanical Properties and Degradability of Biodegradable Polymers

*Jian Zhu, Anil Kumar, Pin Hu, Christoph Habel, Josef Breu, Seema Agarwal**

Table S1. Mechanical properties of PLA/Hec composite films

Samples	Thickness (μm)	Contents of Hec (%)	Strength (MPa)	Modulus (MPa)	Strain at break (%)	Toughness (MJ/m^3)
**PLA film**	37	0	28.7±1.7	972±60	4.1±0.3	71.2±11.8
**3L-PLA/PVP-Hec-5**	28	7.2	42.7±0.7	2369±47	1.9±0.1	43.1±2.9
**3L-PLA/PVP-Hec-10**	28	11.1	35.2±2.6	2199±98	1.7±0.1	34.8±2.9
**3L-PLA/PVP-Hec-20**	29	15.4	38.3±4.6	2051±115	1.9±0.3	37±6.8
**5L-PLA/PVP-Hec-10**	34	13.4	50.4±3.7	2238±193	2.4±0.2	62.2±7.2
**9L-PLA/PVP-Hec-10**	56	16.5	58.1±1.8	2059±93	2.9±0.2	87.3±7.2
**3L-PVP-Hec/PLA-5**	28	—	28.9±3.4	1980±252	1.7±0.3	30.5±6.7

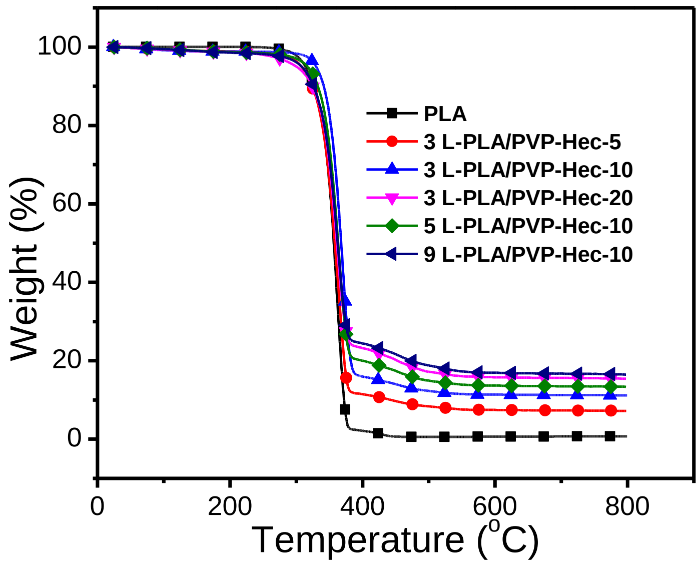

Figure S1. TGA curves of PLA and PLA/PVP-Hec composite films under air atmosphere.

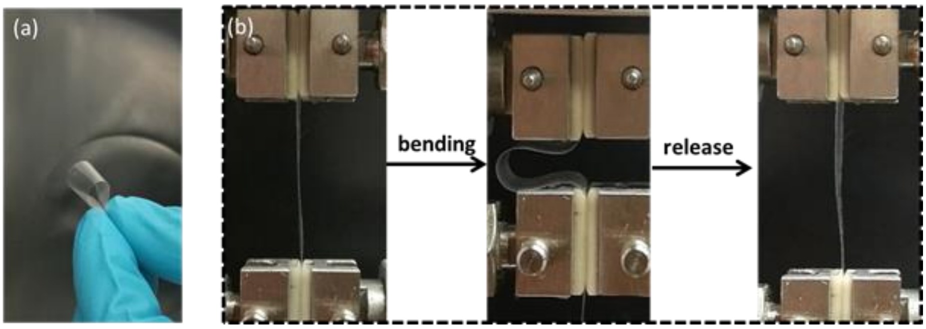

Figure S2. Qualitative bending test of 9L-PLA/PVP-Hec-10 nanocomposite film.

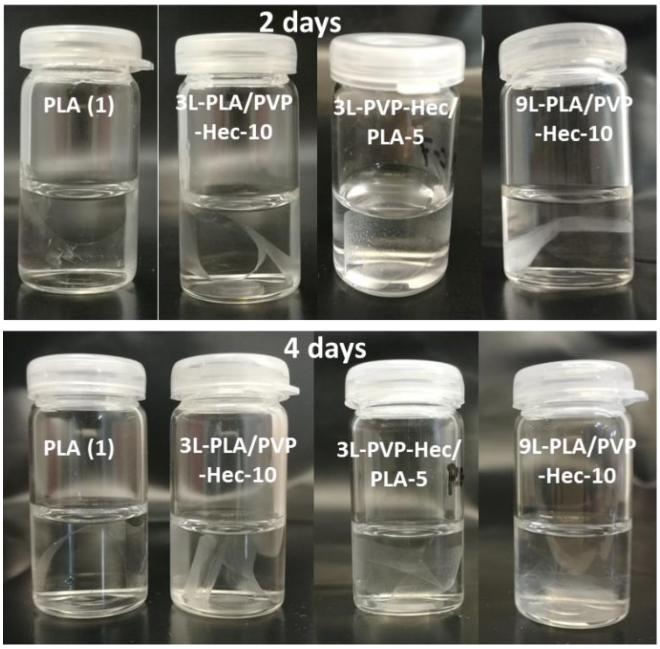

Figure S3. Photos of PLA and PLA/PVP-Hec composite films immersed in enzyme/buffer solution after two and four days.

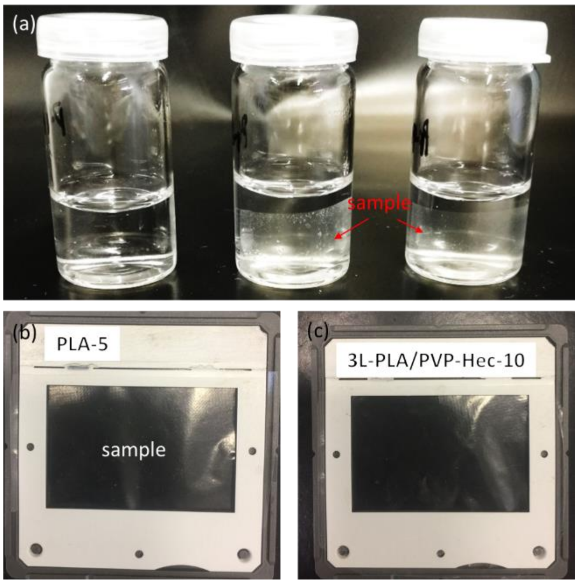

Figure S4. Photos of PLA film and PLA/PVP-Hec composite films for enzyme (a) and compost degradation test (b, c).
